# Loss of β-cell identity and dedifferentiation, not an irreversible process?

**DOI:** 10.3389/fendo.2024.1414447

**Published:** 2024-06-10

**Authors:** Sumit Patel, Maria S. Remedi

**Affiliations:** ^1^ Department of Medicine, Division of Endocrinology, Metabolism and Lipid Research, Washington University School of Medicine, South Euclid Avenue, St. Louis, MO, United States; ^2^ Deparment of Cell Biology and Physiology, Washington University School of Medicine, South Euclid Avenue, St. Louis, MO, United States; ^3^ Center for the Investigation of Membrane Excitability Diseases, Washington University School of Medicine, South Euclid Avenue, St. Louis, MO, United States

**Keywords:** diabetes, type 2 diabetes (T2D), β-cell, identity, dysfunction, dedifferentiation, transdifferentiation, apoptosis

## Abstract

Type 2 diabetes (T2D) is a polygenic metabolic disorder characterized by insulin resistance in peripheral tissues and impaired insulin secretion by the pancreas. While the decline in insulin production and secretion was previously attributed to apoptosis of insulin-producing β-cells, recent studies indicate that β-cell apoptosis rates are relatively low in diabetes. Instead, β-cells primarily undergo dedifferentiation, a process where they lose their specialized identity and transition into non-functional endocrine progenitor-like cells, ultimately leading to β-cell failure. The underlying mechanisms driving β-cell dedifferentiation remain elusive due to the intricate interplay of genetic factors and cellular stress. Understanding these mechanisms holds the potential to inform innovative therapeutic approaches aimed at reversing β-cell dedifferentiation in T2D. This review explores the proposed drivers of β-cell dedifferentiation leading to β-cell failure, and discusses current interventions capable of reversing this process, thus restoring β-cell identity and function.

## Introduction

The international diabetes federation (IDF) determined that approximately 643 million people will have diabetes by the end of 2030 (1). Type 1 diabetes (T1D) is an autoimmune disorder leading to the destruction of the insulin producing β-cells, triggering a dysregulation of glucose homeostasis (2). Type 2 diabetes (T2D) is the commonest form of diabetes accounting for the 90% of the cases. T2D is a metabolic disorder driven by polygenes and environmental risk factors, which is characterized by impaired insulin secretion arising from progressive pancreatic β-cell dysfunction and loss of β-cell mass and insulin resistance in the target peripheral tissues, resulting in multiple long-term health complications (3–5). It had been widely accepted that the progressive decline in β-cell mass in T2D was due to increased apoptosis (6), however, apoptosis alone is not sufficient to explain the marked loss of functional β-cell mass (7–9), suggesting other causes. It is now known that chronic metabolic stress in T2D can lead to loss of β-cell identity with β-cell dedifferentiation, transdifferentiation or degranulation ultimately resulting in β-cell dysfunction (10–12). In this review, we discuss the findings regarding β-cell dedifferentiation and transdifferentiation in diabetes, and evidence supporting prevention and even reversion of these processes leading to restoration of β-cell identity and function.

## Loss of β-cell identity and dedifferentiation in diabetes

Beta-cell dedifferentiation involves loss of mature β-cell identity and expression of markers of islet progenitors and other normally repressed (“disallowed”) genes, which may be one of the driving factors of loss of functional β-cell mass in diabetes. Disallowed genes such as lactate dehydrogenase (*LdhA*) and monocarboxylate transporter-1 (*Mct1*) that are highly expressed in other tissues for non-oxidative glucose metabolism are weakly expressed in the β-cells to prevent abnormal insulin secretion (13, 14). Upregulation of *LdhA* and *Mct* isoforms has been shown in the islets of rodent models of hyperglycemia and diabetes (15–20). Acyl-CoA thioesterase (*Acot7*) that encodes for the enzyme that catalyzes the hydrolysis of long-chain acyl-CoA esters into free fatty acids and coenzyme A, is another β-cell disallowed gene (14, 21). Increased expression levels of *Acot7* have been shown in laser microdissected β-cell–enriched tissue from patients with T2D (22) and in Zucker diabetic fatty rat islets (23). Overexpression of mitochondrial *Acot7* in β-cells of adult mice impaired insulin secretion worsening glucose tolerance (14). Therefore, altered metabolism through altered expression of forbidden genes in diabetes also drives β-cell dysfunction.

The notion that β-cell identity, rather than β-cell apoptosis, may be compromised was first shown in rats exposed to chronic hyperglycemia (15). Additional studies in rodent, non-human primates and human have furthered supported the notion that loss of β-cell identity and dedifferentiation underlies β-cell failure in diabetes (10, 20, 24–34). Although human diabetes studies are limited to terminal endpoints and are reliant on immunofluorescence techniques, β-cell dedifferentiation is more evident than apoptosis (24, 27, 29). Loss of β-cell identity in human T2D samples was shown by reduction of mRNA and protein levels of key β-cell identity markers: MAF BZIP Transcription Factor A (*MafA*) and NKX6 Homeobox 1 (*Nkx6.1*) and pancreatic and duodenal homeobox 1 (*Pdx1*) (27). These findings were further supported by another study that showed that isolated human T2D islets exhibited a similar reduction of mRNA levels of β-cell identity markers: Forkhead Box O1 (*FoxO1*), *MafA* and *Nkx6.1*. Further, immunostaining of T2D pancreata showed β-cells with increased Aldehyde dehydrogenase 1A3 (ALDH1A3) reactivity, indicative of dedifferentiation (24). Single-cell RNA seq (scRNA-Seq) studies from islets of non-diabetic and T2D individuals revealed that α- and β-cells from T2D exhibit similar transcriptomic profiles to islets from juvenile donors (35, 36). Polycomb repressive complex 2 (PRC2), has been identified as an essential chromatin regulatory complex involved in defining cell fate trajectories by repressing transcription and maintenance of β-cell identity. Loss of PRC2 function is observed in islets from human T2D. Elimination of PRC2 in mouse β-cells triggered progressive β-cell dedifferentiation (37). This suggests that disruption of the global gene silencing machinery in β-cells drives β-cell dedifferentiation in diabetes.

**Figure 1:**
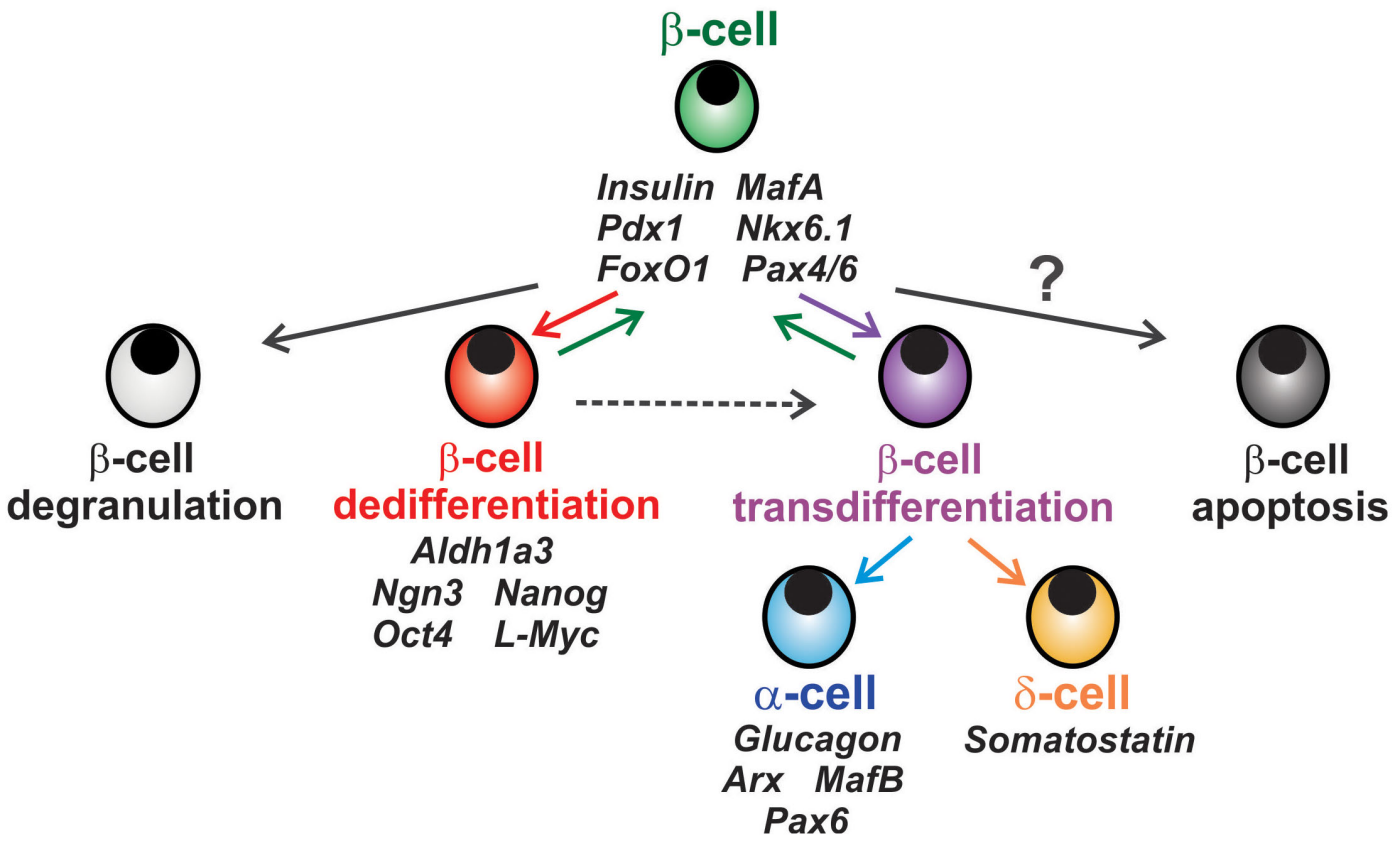
Fates of β-cell in diabetes. Chronic metabolic stress in diabetes can lead to loss of β-cell insulin granules known as β-cell degranulation (light grey cell). β-cells can also incur loss or reduction of mature β-cell identity markers and convert to an endocrine or pancreatic progenitor-like state known as β-cell dedifferentiation (red cell), a process which can be reversible. β-cells can also transition to a different endocrine cell subtype in a process called β-cell transdifferentiation (purple cell) to α- or δ-cells (blue and orange, respectively). The notion that β-cells can undergo apoptosis (dark grey cell) remains controversial, as β-cell apoptosis remains relatively low.

Animal models overcome the limitation of lineage-tracing in human studies, and have shown that β-cells do not undergo apoptosis but rather lose their mature β-cell identity and revert to progenitor-like state through dedifferentiation (10, 26, 28, 32–34). Early studies in mice deficient of *FoxO1* in β-cells under chronic metabolic stress exhibited a marked decrease in β-cell mass due to loss of β cell identity markers such as *MafA* and *Pdx1*, accompanied by dedifferentiation of β-cells into progenitor-like cells expressing Neurogenin3 (*Ngn3*), *Oct4*, *Nanog*, and *L-Myc* (10). Loss of β-cell identity and dedifferentiation was also evident in leptin receptor deficient (*db/db*) and insulin-resistant diabetic (GIRKO) mice, suggesting β-cell dedifferentiation as a cause of β-cell dysfunction in diabetes (10, 38) (**Figure 1**). Beta-cell-specific inactivation of NKX6.1, another marker of β-cell identity, in mice also showed increased expression of *Ngn3* (39). Loss of Urocortin 3 (*Ucn3*), signifying an early trigger of dedifferentiation, was seen in pancreata from obese diabetic (*ob/ob* and *db/db*) mice and from insulin-dependent diabetic mice (*Ins2^Akita^*) (33). Interestingly, islets from *ob/ob*, *db/db* and *Ins2^Akita^* diabetic mice treated with a TGFβ pathway inhibitor Alk5 inhibitor II, demonstrated increased mRNA levels of *Ucn3, MafA*, *Nkx6.1* and *Pdx1*. β-cell dedifferentiation has been reported in non-obese diabetic (NOD) mouse model of T1D (40, 41). A subpopulation of β-cells (~15%) in NOD mice that evaded early immune attack exhibited decreased mRNA levels of β-cell identity markers: *Ins1*, *Ins2*, *Glut2*, *FoxO1*, *Nkx6.1* and *Pdx1*, and increased levels of dedifferentiation markers *Ngn3* and ALDH (40). IRE1α is a kinase involved in triggering unfolded protein response (UPR) due to elevated endoplasmic reticulum stress. NOD mice exhibit elevated UPR response, and β-cell specific deletion of IRE1α in NOD mice resulted in decreased mRNA and protein expression levels of β cell maturity markers *MafA* and *Ucn3.* It was further shown from scRNA-seq and bulk RNA-seq that β cell specific deletion of IRE1α increased expression of *Aldh1a3*, *Gastrin* (*Gast*), and *Ngn3*, indicative of dedifferentiation as a protection against immune detection (41). Treating human β-cells with polyinosinic-polycytidylic acid (PolyI:C), which mimics viral infections contributing to the development of T1D, resulted in decreased expression of β cell–specific genes such as *Ins*, *MafA*, and *Slc30A8*, along with a marked increase in progenitor markers such as *Sox*9, *Hes1*, and *Myc* (42).

ALDH1A3 is an enzyme primarily involved in the catalysis of the oxidation of all-trans retinal to retinoic acid (RA) during RA synthesis (43). ALDH1A3 is a marker that is abnormally expressed in various cancers (44). Murine and human progenitor cells demonstrated increase in ALDH levels in comparison to other hematopoietic cells (45). As previously mentioned, dedifferentiated β-cells also exhibit a progenitor-like state that is similar to the differentiation observed in tumor progression (24, 26). ALDH1A3 was recently shown as a marker of β-cell dedifferentiation, with increased levels in failing β-cells from β-cell-specific *FoxO1* knockout mice, with ALDH1A3 positive cells being less glucose responsive and demonstrating increased markers of uncommitted endocrine progenitors (*Pax6*, *Rfx6*, *Rfx7*, and *Mlxip*l) and decreased levels of mature β-cell markers (*Glucokinase* and *MafA*) (25). It was also demonstrated that while ALDH1A3 positive cells were almost undetectable in islets from pancreatic organ donors without diabetes, it was three-fold higher in islets from T2D individuals despite of adequate glucose control (24, 46). *Db/db* mice exhibit increased number of β-cells expressing ALDH1A3, compared to controls (30). In addition, ALDH1A3 protein expression was significantly elevated in β-cells of C57BL/6J mice subjected to high fat diet (HFD) for 13 weeks compared to 6 weeks on HFD (47).

Remarkably, mice lacking SUR1 (*Abcc8*) subunit of the K_ATP_ channels in β-cells showed increased ALDH1A3 expression, indicative of β-cell dedifferentiation potentially caused by chronically elevated intracellular Ca^2+^ (48). Moreover, *Abcc8* knockout mice subjected to HFD exhibited upregulation of *Aldh1a3* (49), suggesting that chronic β-cell depolarization coupled with overnutrition drives β-cell dedifferentiation. K_ATP_-GOF mouse model of human neonatal diabetes also demonstrated increased ALDH1A3 expression in β-cells, potentially due to hyperstimulated glucose metabolism (50). Indeed, decreasing glucose metabolism by genetic reduction of glucokinase (*Gck*), reduced β-cell ALDH1A3 to control levels (50). Moreover, β-cell specific deletion of microRNA-483 (miR-483) in mice subjected to HFD led to impaired glucose homeostasis and increased β-cell ALDH1A3 expression (51), suggesting that microRNAs may play a critical role in protecting β-cell function by repressing dedifferentiation. Increased ALDH1A3 expression levels accompanied by decreased expression of CHGA (Chromogranin A) and PDX1 was also reported in β-cells of HFD/STZ (streptozotocin)-induced T2D and *db/db* mice. ScRNA-seq of β-cells from multiple-low-dose model of STZ-induced diabetic mice revealed a subset of β-cells with low expression of β-cell identity transcription factors such as *Pdx1*, *Nkx2.2*, *Nkx6.1*, *Pax6*, *Isl1* and *NeuroD1*, and increased *Aldh1a3* (52).

## β-cell dedifferentiation: a reversible process?

Early introduction of intensive insulin therapy in patients newly diagnosed with T2D has showed attainment of long-term remission in approximately 50% of patients, indicating a rescue of β-cell function from glucotoxicity (53). Moreover, a recent report has shown that early short-term insulin intervention coupled with metformin (biguanide) in newly diagnosed T2D patients improved β-cell function with superior and longer lasting glycemic and lipid control compared to glimepiride (sulfonylurea) coupled with metformin (54). Further, transgenic mouse models of monogenic neonatal diabetes with activated K_ATP_ channels resulting in hypoinsulinemia and hyperglycemia also showed loss of β-cell identity and dedifferentiation, evidenced by decreased in *Ins*, *Nkx6.1*, *Pdx1* and a marked increase in *Ngn3* expression (20, 55). Interestingly, these mice not only exhibited normalization of blood glucose levels but also redifferentiation of the same dedifferentiated NGN3-positive cells into insulin-positive mature β-cells upon intensive insulin therapy (20). Notably, neither treatment with insulin, phloridzin nor rosiglitazone could reduce β-cell ALDH1A3 expression, but calorie restriction was able to curtail β-cell dedifferentiation in *db/db* mice (30). Interestingly, infusions of human umbilical cord-derived MSCs (UC-MSCs) in HFD/STZ and *db/db* mice at an early stage of diabetes prevented β-cell dedifferentiation and protected β-cell function by increasing expression levels of CHGA and PDX1 (56). ALDH1A3 protein and mRNA levels were upregulated in islets upon disrupting the activity of CNOT3 in β-cells, an important post-transcriptional regulator of β-cell maturation and identity (57). Furthermore, *db/db* mice with specific deletion of *Aldh1a3* in β-cells showed improved glucose homeostasis, enhanced glucose tolerance paralleled by improved β-cell function, and increased expression levels of PDX1, NKX6.1, E-Cadherin and MAFA (58). To further show that β-cell dedifferentiation can be curtailed, a recent report demonstrated enhanced β-cell function, enhanced glucose tolerance and increased expression of Insulin and PDX1 in *db/db* mice treated with an ALDH1A3 inhibitor (KOTX1) (34). Moreover, recent studies demonstrated loss of β-cell identity with reduction of NKX6.1 and PDX1 expression, and increased ALDH1A3 gene and protein levels in KK and KKA^y^ polygenic mouse models of T2D. Strikingly, KK and KKA^y^ diabetic mice subjected to intermittent fasting for 16 weeks demonstrated enhanced NKX6.1 and PDX1 expression, and decreased ALDH1A3, suggesting protection from loss of β-cell identity in T2D by intermittent fasting (20). Intermittent fasting also showed improved glucose tolerance especially in the severe polygenic KKA^y^ model of T2D. Together, these findings further strengthen that β-cell dedifferentiation is a reversible process.

## β-cell transdifferentiation: another crisis in diabetes?

In addition to dedifferentiation, β-cells have also been shown to transdifferentiate into α-, δ- or PP cells. Increased α/β-cell ratio has been reported in human T2D studies (59–61), with the increased attributed to decreased β-cell mass with unchanged α-cell mass (61–63). *Ex vivo* studies of human islets showed that β-cells after undergoing degranulation can transdifferentiate into α-cells, with expression of PDX1 and NKX6.1 (11). Moreover, conversion of β-cells to α-cells can be curtailed by knockdown of Aristaless-related homeobox (*Arx*), an α-cell lineage marker. The presence of NKX6.1^+^GCG^+^INS^−^ cells in macaques and humans with diabetes was indicative of that loss of functional β-cell mass could partly be due to conversion of β-cells to α-cells (64).

Beta-cell specific deletion of FoxO1 led to β-cell dedifferentiation as discussed above, but also transdifferentiation into α- and δ-cells (10). Overexpression of Paired Box 4 (*PAX4*) in mouse α-cells led to their transdifferentiation into β-cells (65), and inactivation of *Arx* in mouse α-cells led to conversion of α-cells to β-cells expressing key β-cell identity markers such as *Pdx1, MafA* and *Glut2* (66, 67). Mutation of the NK2 homeobox 2 (*Nkx2.2*) tinman (TN) domain or deletion of DNA-methyltransferase 3A (*DNMT3a*) in mouse β-cells caused *Arx*-dependent β-to-α-cell transdifferentiation (68). Deletion of *Nkx2.2* in adult β-cells also demonstrated transdifferentiation into α- or δ-cells (69). Simultaneous deletion of *Arx* and *Dmnt1* (DNA-methyltransferase 1) in mouse α-cells promoted conversion into functional β-cells (70). Meanwhile, ectopic expression of *Arx* in embryonic and adult β-cells led to their transdifferentiation into α- and PP cells (71). Recent findings have shown that X-box binding protein 1 (XBP1), an important regulator of the ER stress response in β-cells also plays a role in maintaining β-cell identity, with inactivation of *Xbp1* in adult mouse β-cells leading to β-cell dedifferentiation, β-to α-cell transdifferentiation and increased α cell mass (72). Therefore, based on the findings, β-cell transdifferentiation may also play an important role in β-cell dysfunction in diabetes.

## Conclusions and perspectives

Compelling evidence of β-cell dedifferentiation and transdifferentiation in diabetes demonstrates plasticity of β-cells and that these processes can be circumvented to restore functional β-cell mass. Recent findings of converting dedifferentiated cells into functional β-cells, either genetically or pharmacologically, provide insights into restoring β-cell identity and function during progression of diabetes. Future studies with advances in omics exploring the differences between dedifferentiated β-cells (ALDH1A3^+^ cells) and endocrine progenitor cells will help to identify potential mechanisms and new targets for preventing β-cell dedifferentiation. This will further enhance our understanding of how ALDH1A3 suppresses β-cell identity through epigenetic and transcriptional regulation. Moreover, identifying subpopulations of β-cells that are susceptible to metabolic stress and vulnerable to dedifferentiation and/or transdifferentiation, versus β-cells “resistant” to metabolic stress could be critical for preventing or even reverting loss of β-cell identity and function in diabetes. A major strength of this review is the comprehensive discussion of the latest studies in rodents demonstrating loss of β-cell identity, dedifferentiation, and transdifferentiation in diabetes. However, a significant limitation stems from the scarcity of human studies addressing loss of β-cell identity and dedifferentiation, mainly due to the limited availability of human pancreatic samples. Consequently, only a handful of reports using human samples are covered in this review.

## Author contributions

SP: Writing – original draft, Writing – review & editing. MR: Writing – original draft, Writing – review & editing.
